# Editorial: Application of artificial intelligence in improving immunotherapeutic efficacy

**DOI:** 10.3389/fphar.2022.1100837

**Published:** 2022-12-13

**Authors:** Jie Li, Yuyuan Zhang, Zaoqu Liu, Xinwei Han

**Affiliations:** ^1^ Department of Interventional Radiology, The First Affiliated Hospital of Zhengzhou University, Zhengzhou, Henan, China; ^2^ Interventional Institute of Zhengzhou University, Zhengzhou, Henan, China; ^3^ Interventional Treatment and Clinical Research Center of Henan Province, Zhengzhou, Henan, China

**Keywords:** artificial intelligence, cancer treatment, immunotherapeutic efficacy, clinical management, predictive tools, translational pharmacology

**FIGURE 1:**
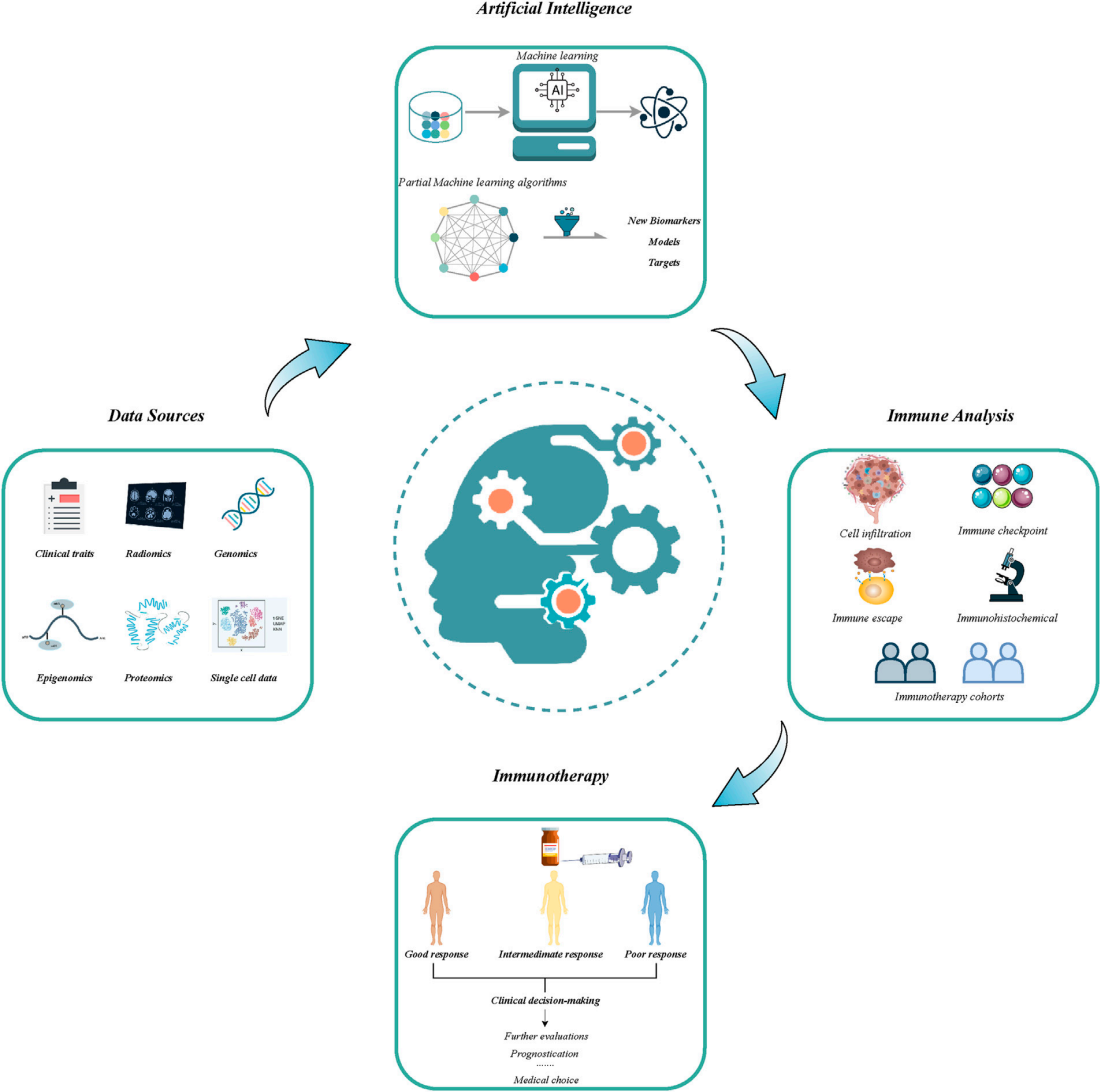
The flowchart of application of artificial intelligence in developing new models and biomarkers for improving immunotherapeutic efficacy.

The advancement in immunotherapy has opened a new epoch in cancer therapy (Wang et al., 2021). However, only subsets of patients have yielded considerable benefits from immunotherapy (Sharma et al., 2021). The unsatisfactory efficacy may primarily arise from the restricted understanding of resistance mechanisms, lack of robust biomarkers, and unavoided immune-related adverse events. Thereby, new assessment approaches are imperative to optimize immunotherapy and improve clinical outcomes. With advancements in high-throughput sequencing techniques and computational biology, numerous predictive tools combining clinical and molecular traits displayed excellent performance to robustly address the classification of tumor types, prediction of prognosis, and immunotherapy response (Wang et al., 2022a; Liu Z. et al., 2022). This Research Topic assembled eight original research articles, which applied artificial intelligence to develop novel targets, drugs, and immunotherapy predictive markers, from large-scale data, and provided excellent paradigms on how to exploit artificial intelligence to generate reliable predictive tools (Figure 1).

Despite immune checkpoint inhibitors (ICIs) exhibiting durable responses and prolonged survival across multiple tumors, lower response rates, higher resistance rates, and costs hinder clinical utilization (You et al., 2020), which prompts researchers to develop novel biomarkers predicting the efficacy of immune checkpoint treatment (ICT). Currently, numerous biomarkers including microsatellite instability (MSI) (André et al., 2020), programmed death-ligand 1 (PD-L1) expression (Lin et al., 2018), and tumor mutational load (TMB) (Samstein et al., 2019) have been proposed to identify susceptibility to ICT. Nevertheless, previous studies reported no significant correlation between these biomarkers and ICT response, and some are even contradictory (Wu et al., 2019; Bai et al., 2020). Complex mechanisms of anti-tumor immune response and immune escape limit the functionality of a single biomarker (Wang et al., 2022b). To address the limitations of single biomarkers, the current studies focused on integrating different types of data to develop comprehensive predictive tools for immunotherapy.

Recent evidence reveals that non-apoptotic regulated cell death (RCD), encompassing autophagy, pyrolysis, and necrosis, displays synergistic anti-tumor immune responses. Thus, the development of biomarkers against non-apoptotic RCD may improve the efficacy of immunotherapy (Clarke and Simon, 2019; Gong et al., 2019; Wang et al., 2020). Pyroptosis, a lytic and programmed inflammatory cell death pathway distinct from apoptosis, has been shown to enhance immune activation and function (Gao et al., 2022). In the research by Ma et al.; Qi et al., pyroptosis-related biomarkers were developed with bioinformatics technology, effectively stratifying patients with distinct prognoses and deciphering the relevance of pyroptosis to immunotherapy. Necrosis as a mode of immunogenic cell death with tight crosstalk with anti-tumor immunity may elicit and amplify antitumor immunity in cancer therapy (Gong et al., 2019). Meng et al. constructed a necrosis-related lncRNA prognostic signature (NLPS) and comprehensively analyzed the immune and pharmacological landscape of NLPS to guide clinical decision-making in the management of Head and neck squamous cell carcinoma (HNSCC). These results indicated that the development of new biomarkers and drugs targeted cell death modes may provide novel insights to optimize the selection of patients for ICT and clinical benefits.

Additionally, remodeling of the immune microenvironment (TIME) provides immunologically permissive conditions for tumor progression and immune escape. With the influence of multiple regulators, immune cells (proportions and quantities) and immune molecules become altered (Zhang et al., 2020; Mao et al., 2021), and deciphering this heterogeneous variation in the immune landscape throws light on the development of potential targets and biomarkers. Recently, long non-coding RNAs (lncRNAs) were detected to facilitate cancer progression and immunotherapy resistance by regulating tumor microenvironments (TME), such as the proportion of effector to regulatory T-cell, antigen presentation, and immune escape (Denaro et al., 2019). Liu et al. comprehensively investigated the relationship between tumor proliferation and lncRNA and developed a proliferation-related lncRNA-related signature that could serve as a promising tool to optimize immunotherapy decision-making. In another study, Gou et al. decoded the TME *via* estimating the TME infiltration patterns of various patients, and identified different subtypes of TME, further they utilized machine learning algorithms to generate TME-related scores to enhance the understanding of TME and guide immunotherapy strategies. Furthermore, Xia et al.; Zhao et al. constructed the predictive models based on lysyl oxidase family enzymes (LOXs) and N6-methyladenosine (m6A) epigenetic modification, respectively, investigated the prognostic value, and immune landscape, highlighting the promise of extracellular proteins and epigenetic modifications in cancer immunotherapy. Of note, Mao et al. systematically established links between senescence-related genes (SRGS) signature and TIME, and prognosis, providing a stable tool for detecting high-risk patients and further improving clinical outcomes. Collectively, targeting the heterogeneous TIME might facilitate the generation of novel biomarkers and predictive tools to improve the response to immunotherapy.

This Research Topic “*Application of artificial intelligence in improving immunotherapeutic efficacy*” served as an attractive platform for developing novel predictive biomarkers for immunotherapy. Although the articles under this topic may not cover the current research in immunotherapy prediction, we (the editors) believe that novel models and biomarkers for immunotherapy using large-scale data and artificial intelligence will be promising approaches to optimize decision-making and surveillance protocols for individual patients across multiple cancer types.
